# Association between the Mode of Delivery and Vertical Transmission of Human Papillomavirus

**DOI:** 10.3390/v16020303

**Published:** 2024-02-16

**Authors:** Émilie Nantel, Marie-Hélène Mayrand, François Audibert, Joseph Niyibizi, Paul Brassard, Louise Laporte, Julie Lacaille, Monica Zahreddine, William Fraser, Diane Francoeur, Marie-Josée Bédard, Isabelle Girard, Jacques Lacroix, Ana Maria Carceller, François Coutlée, Helen Trottier

**Affiliations:** 1 Department of Social and Preventive Medicine, Université de Montréal, Montreal, QC H3N 1X9, Canada; emilie.nantel.cissslan@ssss.gouv.qc.ca (É.N.); marie-helene.mayrand@umontreal.ca (M.-H.M.); joseph.niyibizi@umontreal.ca (J.N.); monica.zahreddine@umontreal.ca (M.Z.); 2 Sainte-Justine Hospital Research Center, Montreal, QC H3T 1C5, Canadajlacroix052@gmail.com (J.L.); anacarceller@hotmail.com (A.M.C.); 3 Centre de Recherche du CHUM (CRCHUM), Montreal, QC H2X 0A9, Canada; julie.lacaille.chum@ssss.gouv.qc.ca (J.L.); marie-josee.bedard@umontreal.ca (M.-J.B.); francois.coutlee.med@ssss.gouv.qc.ca (F.C.); 4 Department of Obstetrics and Gynecology, Université de Montréal, Montreal, QC H3N 1X9, Canada; 5 Department of Obstetrics and Gynecology, Sainte-Justine Hospital, Montreal, QC H3T 1C5, Canada; francois.audibert@umontreal.ca; 6 Division of Clinical Epidemiology, McGill University Health Center, Montreal, QC H4A 3J1, Canada; paul.brassard@mcgill.ca; 7 Department of Obstetrics and Gynecology, Centre de Recherche du CHUS, Université de Sherbrooke, Sherbrooke, QC J1H 5N4, Canada; william.fraser@usherbrooke.ca; 8 Department of Obstetrics and Gynecology, St-Mary’s Hospital Center, Montreal, QC H3T 1M5, Canada; 9 Department of Pediatrics, Division of Pediatric Intensive Care Medicine, Sainte-Justine Hospital, Montreal, QC H3T 1C5, Canada; 10 Department of Pediatrics, Université de Montréal, Sainte-Justine Hospital, Montreal, QC H3T 1C5, Canada; 11 Departement of Microbiology, Infectiology and Immunology, Université de Montréal, Montreal, QC H2X 0A9, Canada

**Keywords:** human papillomavirus (HPV), vertical transmission, pregnancy, mode of delivery, vaginal delivery, caesarean section

## Abstract

Human papillomavirus (HPV) can be vertically transmitted. Our objective was to measure the association between the mode of delivery and the detection of HPV in infants. We used data collected from pregnant women during the HERITAGE study. Self-collected vaginal samples from the first and third trimester were obtained for HPV testing. Specimens from oral, pharyngeal, conjunctival and anogenital mucosa were collected from infants 36–48 h after delivery and at 3 months of age. All samples were tested for HPV DNA by the Linear Array assay. Adjusted odd ratios (aOR) and 95% confidence interval (CI) were estimated using multivariate logistic regressions. From the 282 women revealed to be HPV-positive in both the first and third trimesters, 25 infants were born HPV-positive. The overall probability of transmission was 8.9% (25/282); 3.7% (3/81) in participants with a caesarean section and 10.9% (22/201) for those who delivered vaginally. Vaginal delivery increased the risk of HPV in infants compared to caesarean (aOR: 3.63, 95%CI: 1.03–12.82). Infants born after a caesarean with ruptured membranes were not at increased risk of HPV compared to infants born after an elective caesarean section with intact membranes (aOR: 1.31, 95%CI: 0.10–17.76). Our results support the hypothesis that transmission occurs mostly during the passage in the vaginal canal.

## 1. Introduction

Human papillomavirus (HPV) is the most common sexually transmitted infection in the world [1,2]. The causal role of oncogenic, or high-risk (HR), HPVs in anogenital and oral cancers is now well established [3]. Other low-risk (LR) HPVs are responsible for anogenital warts [4,5] and recurrent laryngeal papillomatosis [5,6], a rare condition that can cause severe respiratory problems. HPVs are mainly transmitted sexually [7], although other modes of transmission exist [8]. Current evidence suggests that HPV can be transmitted vertically [7], probably through a variety of mechanisms, such as prenatal transmission, from amniotic fluid or placenta, or perinatal transmission, from ascending affection after rupture of membranes or during the passage through the birth canal. Only a few studies have focused specifically on the risk factors associated with HPV perinatal transmission, such as the mode of delivery [9,10,11,12,13,14,15,16,17,18,19,20,21,22], and conflicting results have been reported. These studies are summarized in the meta-analysis published by Chatzistamatiou et al. [23], which includes a total of 446 mother–child pairs (141 caesarean deliveries and 305 vaginal deliveries) and showed that, even if HPV was detected in nearly 15% of infants born to caesarean section, caesarean significantly reduces the risk of vertical transmission of HPV compared to vaginal delivery (14.9% versus 28.2%, OR: 0.515, 95% CI: 0.34–0.78. Our objective was to measure the independent association between the mode of delivery and the detection of HPV DNA in infants in a large prospective cohort study of pregnant women.

## 2. Materials and Methods

### 2.1. Participants

We used data from 1052 pregnant women enrolled as participants in the HERITAGE (Human Papillomavirus perinatal transmission and risk of HPV persistence among children) prospective cohort study.. The design of HERITAGE has been published previously [24]. Briefly, recruitment took place in two phases (2010–2012, 2015–2016) in Montreal (Canada) in three hospitals and their affiliated clinics: Centre Hospitalier Universitaire Sainte-Justine, Centre Hospitalier de l’Université de Montréal, and Saint-Mary’s Hospital Center. Inclusion criteria were being 18 or older, being 6–14 weeks pregnant and planning on giving birth at one of the participating hospitals. Exclusion criteria were HIV infection and the inability to provide informed consent. Pregnant women were followed up with their offspring until the children were 2 years old.

**Figure 1:**
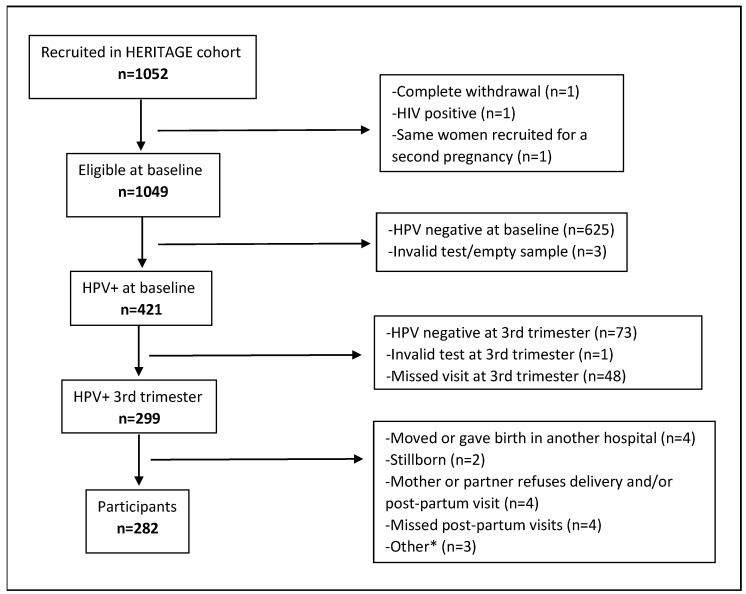
Study flow diagram. Abbreviations and symbols: HPV: Human Papillomavirus; HIV: human immunodeficiency viruses. *: termination of pregnancy (n = 1), baby needs special follow-up (n = 1), too much stress for the baby (n = 1).

In this analysis, we included HERITAGE participants who (1) had a positive HPV test at enrollment, (2) had a second positive HPV test at their third trimester visit, (3) had a live birth with available delivery data, and (4) had completed the newborn and/or three-month visit. There were 282 participants who fulfilled these criteria (Figure 1).

### 2.2. Data Collection

Participants self-collected vaginal samples for HPV DNA testing. The first sample was collected at enrollment (6–14 weeks of pregnancy) and those who tested positive collected a second sample during the third trimester (32–35 weeks of pregnancy). Both samples were collected by the women using a dry Dacron swab (Copan Italia S.p.A., Brescia, Italy). They were instructed to insert the swab gently in the vaginal canal, at least 2.5 inches, and rotate three times. Specimens were individually rinsed in a plastic vial using 1.5 mL of PreservCyt (Cytyc Corporation, Boxborough, MA, USA). The DNA was then purified according to the Master pure procedure and stored frozen at −80° awaiting HPV testing that was performed in batches.

In infants, specimens from the buccal, pharyngeal and anogenital mucosa (labia and anal region, or prepuce and scrotum and anal region) were collected by a nurse using a dry Dacron swab, and specimens were collected from the conjunctival mucosa in each eye using a soft swab (FLOQSwabs, Copan Flock Technologies, Brescia, Italy). The nurse collected samples 36 to 48 h after birth to decrease the detection of maternal HPV that could have been deposited at delivery, and at three months old. Specimens were rinsed and stored as described for vaginal samples. Conjunctival swabs for both eyes were stored in the same vial.

Questionnaires were used to collect data at enrollment, including socio-demographic data (age, ethnicity, relationship status, education, household income), medical history data (parity, cervical cytology results, history of genital warts and abnormal cervical cytology, HPV vaccination) and behavioral and sexual activity data (drug use, alcohol consumption, smoking history, number of sexual partners in the past year, age of first sexual intercourse). A second questionnaire, at delivery, collected updated information on drug use, alcohol consumption, smoking, changes in medical history and sexual activity during pregnancy. Medical files were reviewed to collect data on delivery related variables (mode of delivery, time of membrane rupture, time of birth and reason for caesarean section).

### 2.3. HPV Testing and Genotyping

All samples were analyzed using the Linear Array assay (Roche Molecular Systems, Branchburg, NJ, USA) for detection and genotyping of 36 mucosal HPV genotypes, including HPV-6, 11, 16, 18, 26, 31, 33, 34 (formerly known as 64), 35, 39, 40, 42, 44 (formerly known as 55), 45, 51, 52, 53, 54, 56, 58, 59, 61, 62, 66, 67, 68, 69, 70, 71, 72, 73, 81, 82, 83, 84 and 89. β-globin DNA was co-amplified to assess DNA integrity and to screen for the presence of inhibitors. We used negative, weak positive and strong positive as controls in each amplification run. Samples negative for β-globin and HPV were considered invalid. We used extensive safeguards to avoid contamination. The probe used for the detection of HPV-52 also reacts to the presence of HPV-33, 35 and 58. In case of a reaction in the HPV-52 probe, samples were further tested using a HPV-52 specific real-time PCR assay [25].

### 2.4. Statistical Analysis

We used proportions, medians and interquartile ranges (IQR), means and standard deviations (SD) to describe the study population and the prevalence of type-specific HPV in participants. The association between the mode of delivery and HPV detection in infants was measured using logistic regressions.

Three sets of regression analysis were completed considering different definitions of exposure. In the first set, the mode of delivery was considered as a binary exposure variable comparing vaginal delivery to caesarean section. In the second set, we used a 3-category variable comparing vaginal delivery, caesarean section occurring after membrane rupture, and caesarean section with intact membranes. In the third analysis, we explored the role of the duration of membrane rupture (continuous variable). Thirty-eight women who had an elective caesarean section and four women with missing data regarding the duration of membrane rupture were excluded from the third analysis.

A positive outcome was defined as the detection of any HPV DNA in at least one site on the infant (anogenital, conjunctival, pharyngeal or oral mucosa) at least at one of the post-partum visits (birth and/or 3 months). In this study, we considered HR-HPV genotypes 16, 18, 31, 33, 35, 39, 45, 51, 52, 56, 58, 59, 66, 68, 73, 82. These include genotypes (16, 18, 31, 33, 35, 39, 45, 51, 52, 56, 58, 59) that are officially recognized as HR-HPV by the latest classification published by the World Health Organization’s International Agency for Research on Cancer [26] and genotypes 66, 68, 73 and 82 that are included as HR-HPV by other classifications [27]. The remaining genotypes, HPV-6, 11, 26, 34, 40, 42, 44, 53, 54, 61, 62, 67, 69, 70, 71, 72, 81, 83, 84 and 89, were considered LR-HPV [26,27].

Four women enrolled in the study had a multiple pregnancy (three sets of twins and one set of triplets). The HPV outcome for these sets of babies (n = 4) was defined combining data (i.e., detection of HPV DNA in at least one site on at least one baby) and the duration of the membrane rupture was calculated by considering the time at birth of the first baby to be born.

We estimated the crude and adjusted odd ratio (OR) with 95% confidence intervals (CI). We adjusted each logistic regression model for confounding variables using the change in estimate methods [28] with a cut-off of 3% (variables that changed the crude OR by ± 3% were retained in the multivariable model). Based on the literature, the following potential confounding variables measured at recruitment were considered: age (≤25, 26–35, ≥36), ethnicity (Caucasian, other), in a relationship (yes, no), some university education (yes, no), annual household income (≤CAD 39,000, CAD 40,000–59,999, CAD 60,000–99,999, ≥CAD 100,000), smoking status (current, former, never), parity (nulliparity, multiparity), new sexual partner in the past year (yes, no). We also considered the following variable measured during follow-up: gestational age at delivery (in weeks). Mode of delivery (vaginal, caesarean section) was considered as a potential confounder for the third analysis (duration of membrane rupture). Statistical analyses were completed using Stata SE version 13.

## 3. Results

The main characteristics of the 282 HPV-positive study participants are described in Table 1. At enrollment, women were between 19 and 47 years of age, with a median age of 31 years. Most of the participants were Caucasian (78.7%) and had some university-level education (59.2%). About 90% of the women were in a relationship at enrollment and 87.6% of the women did had not had any new sexual partner in the year before. Out of the 282 women, 201 (71.3%) had a vaginal delivery and 81 (28.7%) had a caesarean section.

HPV infection with multiple genotypes was frequent, with 146 women (51.8%) harboring more than one genotype in the first trimester of pregnancy and 122 (43.3%) harboring more than one genotype in the third trimester. The most prevalent genotypes at enrollment were HPV-53 (n = 46; 16.3%), 16 (n = 41; 14.5%) and 62 (n = 41; 14.5%). At the third-trimester visit, these three genotypes remained the most prevalent, with 36 (12.7%), 35 (12.4%) and 31 (11.0%) cases, respectively.

A total of 25 out of 282 infants tested positive for HPV DNA at birth or three months of age (probability of transmission: 8.9%, 95% CI: 5.8–12.8%). Table 2 describes, for each HPV-positive infant, the detected HPV genotypes, the site of detection and the genotypes detected in their mother during pregnancy. At the first infant visit (36–48 h after birth), 21 newborns were HPV-positive and 27 genotypes were detected. At second infant visit (3 months), five infants tested positive and five genotypes were detected. Only one baby tested positive at both visits, though for two different genotypes, both of these genotypes being concordant with the mother’s. At the first infant visit, 14/21 (67%) HPV-positive newborns were positive for at least one HR-HPV genotype. A total of 17 HR-HPV genotypes were detected in infants at the first visit, and 3 were detected at the second visit. Overall, the most prevalent genotypes in infants were HR-HPV genotypes 66 (n = 5), 51 (n = 3), 16 (n = 3) and LR-HPV genotype 62 (n = 3).

In 20 (80%) HPV-positive infants, HPV DNA was detected in only one sampled site (oral, pharyngeal, conjunctival or anogenital mucosa). Five infants had HPV DNA in multiple sites but none of the infants had HPV DNA in all sites. HPV DNA was detected in 11 eye samples, 10 oral samples, 8 anogenital samples and 3 pharyngeal samples. Of the 25 HPV-positive infants, 21 (84%) had a concordant genotype with their mother and all of them were born vaginally. Five out of the twenty-five (20%) HPV-positive infants had more than one genotype detected at birth and none had more than one genotype detected at the 3-month visit.

Among the 25 HPV-positive infants, 3 were born by caesarean section and 22 were born vaginally. The probability of HPV transmission were 3.7% (95% CI: 0.8–10.4%) and 10.9% (95% CI: 7.0–16.1%) following caesarean section and vaginal delivery, respectively (Table 3). We found that vaginal delivery significantly increased the risk of HPV detection in infants compared to caesarean section (adjusted OR: 3.63, 95% CI: 1.03–12.82). The adjusted ORs for HPV transmission after vaginal deliveries and after caesarean section following the rupture of membranes were, respectively, 4.14 (95% CI: 0.46–38.99) and 1.31 (95% CI: 0.10–17.76) when compared to elective caesarean section (intact membranes).

We found no association between duration of membrane rupture (continuous variable, in hours) and the detection of HPV in infants (OR: 1.00, 95% CI: 0.97–1.02). We performed sensitivity analyses to assess the impact of the duration of rupture, looking into a threshold effect treated as a categorical variable using different categorizations. All the results for different categorizations were similar and none were statistically significant (*p* values > 0.05).

## 4. Discussion

A total of 25 out of 282 (8.9%) infants born to mothers who tested positive for HPV in the first and third trimester were found to be HPV positive in our study. This probability is low compared to other studies [9,11,13,15,16,17,20,29]. Among 15 studies on HPV vertical transmission, when restricting the analysis to women who were HPV positive during the pregnancy, the probability of vertical transmission of HPV varied from 3% to 79% [9,10,11,12,13,14,15,16,17,18,19,20,30,31,32], with a mean of 28%. This wide range could be due to heterogeneous sampling methods in women and in infants, contamination of samples, and the use of different HPV DNA detection assays.

In our study, the risk of HPV detection was significantly higher in infants born by vaginal delivery (adjusted OR: 3.63, 95% CI: 1.03–12.82). This is similar to the conclusions from two systematic reviews. Medeiros et al. [33], in 2005, found that the pooled risk of vertical transmission from seven studies [15,16,17,18,19,20,29] was higher in vaginal deliveries compared to caesarean section (RR: 1.8, 95% CI: 1.34–2.43). When considering that HPV transmission occurred only when the same genotype(s) were detected in infants and mothers, a meta-analysis by Chatzistamatiou et al. [23] that included eight studies [9,11,12,14,15,16,17,20] found a pooled probability of vertical transmission of 24.6% (95% CI: 20.5–29.9). This meta-analysis also found a significantly lower risk of HPV transmission in infants born by caesarean section compared to vaginal delivery (RR: 0.52, 95% CI: 0.34–0.78). In our study, of the 25 HPV-positive mother–infant pairs, 21 pairs (84%) were concordant. When restricted to concordant mother–infant pairs, the probability of transmission in our study was 7.4% (21/282), which is lower than the estimates found in the meta-analysis [23]. Also, it is important to note that all HPV positive children born by caesarian section in our study (n = 3) were positive for a genotype that was not concordant with their mothers during pregnancy, thus 100% (21/21) of the HPV positive infants in concordant pairs were born vaginally. It is possible that we did not detect all genotypes in the mother during pregnancy, but also horizontal transmission in the children born by caesarean section cannot be excluded.

Some proposed that vertical transmission could occur not only in the vaginal canal, but also by ascending infection from the maternal genital tract after membrane rupture [11,14,33,34]. However, in our study, caesarean section following the rupture of membranes was not associated with an increased risk of HPV transmission (adjusted OR: 1.31, 95% CI: 0.10–17.76), when compared to caesarean with intact membranes. Moreover, membrane rupture duration was not associated with vertical HPV transmission (OR: 1.00, 95% CI: 0.97–1.02). This is in contrast with the findings of Tenti et al. [16], who reported a significantly lower risk of HPV transmission in infants born less than two hours after the rupture of membranes (0/11) compared to those with longer duration of membrane rupture (11/26, 42%) (*p* = 0.009). However, Tenti et al. [16] might have found a positive association with membrane rupture duration simply because they included women who had an elective caesarean section in the group with the shorter duration of membrane rupture. Park et al. [11] also found no association between the detection of HPV in infants and labour duration (*p* = 0.64) nor premature rupture of membranes (*p* = 0.999).

Our study has many strengths, such as the sampling of many sites in infants, including the conjunctive. Indeed, we found HPV DNA in the eye mucosa of 11 (44%) HPV-positive infants. Twenty of the twenty-five infants (80%) tested positive in only one sampled site. This suggests that sampling only one site in infants, as in some studies [9,11,15,16,20], would have underestimated the real proportion of HPV-positive infants. Another strength of our study is the sample size of HPV-positive pregnant women, which is at least double that of previous studies [9,10,11,12,13,14,15,16,17,18,19,20]. Also, most studies on HPV vertical transmission included HPV-positive and HPV-negative women in their analysis [10,12,13,15,19,20]. This study restricted the sample to HPV-positive pregnant women only, in order to gain a more accurate estimation of the transmission probability. The use of self-collected vaginal samples was also more appropriate than cervical samples in a context of a vertical transmission study, allowing us to sample the entire vaginal canal and not only the cervix.

This study also has some limitations. Perinatal HPV was defined considering the detection of HPV DNA at birth and at the three-month visit. Particularly for infants who were HPV positive at three months only (n = 4), we could not rule out the possibility of post-natal transmission. Horizontal transmission can occur through a nonsexual route, such as inoculation through skin-to-skin and skin-to-mucous membrane contact or contact with contaminated objects [22]. This may also explain some HPV genotype discordance between mothers and their infant. Furthermore, it is also possible that HPV was not detected in participants due to sampling outside the infected mucosal region or infection in another untested mucosa, such as an anal infection. It is also possible that we could have missed an HPV genotype in the mother at birth because the mother was not tested at the time of delivery in our study. Moreover, as only a few cases of HPV 6 and 11 were detected, it was not possible to analyse the association between the mode of delivery and these specific genotypes.

It is important to note that the results of our study do not, on their own, suggest an indication for caesarean section for HPV-positive women, as the benefits and risks must be carefully weighted, especially given that almost all HPV positive infants will clear the infection within 2 years [8,35]. The only documented severe clinical complication of perinatal HPV transmission is respiratory papillomatosis (caused by HPV genotypes 6 and 11), a very rare condition (one to four cases in every 100,000 births [36]). Fortunately, all available HPV vaccines target genotypes 6 and 11, and consequently HPV vaccination programs could essentially eliminate this pathology in the coming decades.

## 5. Conclusions

In conclusion, our results suggest that vertical HPV transmission mostly occurs during the passage through the birth canal, as only a few infants born by caesarean section were HPV positive. Caesarean section considerably reduced the risk of HPV transmission in infants. Since the rupture of membranes before caesarean section and the duration of membrane rupture did not significantly impact the risk of HPV detection in infants, our data more strongly support the mechanism of HPV transmission through the vaginal canal during delivery rather than by an ascending infection after rupture of membranes. One of the important questions is whether HPV detected at birth can have a negative impact on a child’s health. Further studies are needed to answer this question.

## Figures and Tables

**Table 1:** Characteristics of the study participants.

Baseline Characteristics	Vaginal Delivery (n = 201)	Caesarean Section (n = 81)	All Participants (n = 282)
Age			
Median (IQR)	30 (27–33)	32 (29–35)	31 (28–34)
Range	19–43	20–47	19–47
Age category, n (%)			
25 and under	29 (14.4%)	6 (7.4%)	35 (12.4%)
26–35	147 (73.1%)	57 (70.4%)	204 (72.3%)
36 and over	25 (12.4%)	18 (22.2%)	43 (15.3%)
Ethnicity, n (%)			
Caucasian	164 (81.6%)	58 (71.6%)	222 (78.7%)
Other	37 (18.4%)	23 (28.4%)	60 (21.3%)
In a relationship with a partner, n (%)			
Yes	178 (88.6%)	76 (93.8%)	254 (90.1%)
No	23 (11.4%)	5 (6.2%)	28 (9.9%)
Some university education, n (%)			
Yes	123 (61.2%)	44 (54.3%)	167 (59.2%)
No	78 (38.8%)	37 (45.7%)	115 (40.8%)
Annual household income (CAD), n (%)			
≤39,000	33 (17.2%)	11 (14.3%)	44 (16.4%)
40,000–59,999	30 (15.6%)	15 (19.5%)	45 (16.7%)
60,000–99,999	56 (29.2%)	20 (26%)	76 (28.3%)
≥100,000	73 (38%)	31 (40.3%)	104 (38.7%)
Missing	9	4	13
Smoking status, n (%)			
Current	36 (17.9%)	8 (9.9%)	44 (15.6%)
Former	40 (19.9%)	16 (19.8%)	56 (19.9%)
Never	125 (62.2%)	57 (70.4%)	182 (64.5%)
Vaccinated against HPV, n (%)			
Yes	27 (14.3%)	10 (13.3%)	37 (14%)
No	162 (85.7%)	65 (86.7%)	227 (86%)
Missing	12	6	18
Parity, n (%)			
Nulliparity	101 (55.2%)	42 (55.3%)	143 (55.2%)
Multiparity	82 (44.8%)	34 (44.7%)	116 (44.8%)
Missing	18	5	23
Any new sexual partner in the past year, n (%)			
Yes	26 (12.9%)	9 (11.1%)	35 (12.4%)
No	175 (87.1%)	72 (88.9%)	247 (87.6%)
**Characteristics during pregnancy**			
Gestational age, delivery			
Mean (SD)	39.4 (1.37)	38.8 (2.27)	39.3 (1.70)
Median (IQR)	39.6 (38.6-40.4)	39.1 (38-40.4)	39.5 (38.4-40.4)
Range	34.6-41.9	30.7-42	30.7-42
Maternal HR-HPV, n (%)			
Any HR-HPV at 1st trimester	148 (73.6%)	54 (66.7%)	202 (71.6%)
Any HR-HPV at 3rd trimester	147 (73.1%)	49 (60.5%)	196 (69.5%)
Maternal multiple infection, n (%)			
Multiple infection at 1st trimester	100 (49.8%)	46 (56.8%)	146 (51.8%)
Multiple infection at 3rd trimester	81 (40.3%)	41 (50.6%)	122 (43.3%)

HPV: Human papillomavirus; HR-HPV: high-risk Human papillomavirus; LR-HPV: low-risk Human papillomavirus. SD: standard deviation; IQR: interquartile range; CAD: Canadian Dollar.

**Table 2:** HPV genotyping results in mother during pregnancy and in infant for all HPV-positive infants at birth or 3 months of age.

ID	Mode of Delivery	Genotype Detected				Site in Baby *	Concordant Pair
		Mother, Baseline	Mother, 3rd Trimester	Infant, 36–48 h after Birth	Infant, 3 Months		
1	Caesarean section	53	59	84	-	O	No
2	Caesarean section	54	54, 83	82	-	O	No
3	Caesarean section	66	58, 66	73	*MV*	G	No
4	Vaginal	53, 59, 89	53, 89	51	*MV*	E	No
5	Vaginal	89	89	-	89	O	Yes
6	Vaginal	66, 70, 89	66	*MV*	66	O, E	Yes
7	Vaginal	31, 35, 42, 51, 53, 58	31, 35, 42, 51, 53, 58, 84	39, 42	-	O, P, G	Yes
8	Vaginal	31, 40, 45, 54, 59, 67, 81, 83	31, 40, 81, 83, 89	-	89	O	Yes
9	Vaginal	35, 45, 61	35, 45, 89	35	*MV*	G	Yes
10	Vaginal	6, 39	6, 39	6	-	E	Yes
11	Vaginal	39, 59	31, 66	31, 66	-	E	Yes
12	Vaginal	66	66	66	*MV*	P, E	Yes
13	Vaginal	45, 62	45, 62	62	-	G	Yes
14	Vaginal	18	18	18	-	O	Yes
15	Vaginal	51	51	51	-	G, E	Yes
16	Vaginal	40, 42, 71, 83	40, 62, 71, 73	62	-	E	Yes
17	Vaginal	66	66	66	-	E	Yes
18	Vaginal	16, 35, 42, 52	16, 35, 52	16, 35, 52	-	G	Yes
19	Vaginal	39, 66	31, 39, 66	66	-	G	Yes
20	Vaginal	16, 18, 45, 53, 82	16, 45, 53	16, 53	-	O	Yes
21	Vaginal	42, 83, 84, 89	26, 42, 83, 84, 89	83	*MV*	P	Yes
22	Vaginal	35, 44, 52, 56	35, 44, 52, 56, 89	44	52	E	Yes
23	Vaginal	16, 62, 81	16, 62, 81	16, 62	*MV*	O, G, E	Yes
24	Vaginal	34, 53, 89	66, 84, 89	84	-	E	Yes
25	Vaginal	51, 84	51	-	51	O	Yes

* O = oral mucosa, P = pharyngeal mucosa, G= genital mucosa, E = eye mucosa. *MV* = missed visit. -: no HPV DNA detected.

**Table 3:** Association between the mode of delivery and HPV detection in infants.

Exposure	n	Cases of HPV in Infants n (%)	Crude OR (95% CI)	Adjusted OR (95% CI)
Caesarean section	81	3 (3.7)	1	1 ^A^
Vaginal delivery	201	22 (10.9)	3.20 (0.93–10.99)	**3.63 (1.03–12.82)**
Elective caesarean section (intact membranes)	38	1 (2.6)	1	1 ^B^
Caesarean section after rupture of membranes	43	2 (4.7)	1.80 (0.16–20.73)	1.31 (0.10–17.76)
Vaginal delivery	201	22 (10.9)	4.55 (0.59–34.80)	4.14 (0.46–38.99)
Time between rupture of membranes and birth	240 *	24 (10.0)	1.00 (0.97–1.02)	- ^C^

Bold values represent statistically significant results; HPV: Human papillomavirus; OR: odds ratio; CI: confidence interval. *: 38 women who did not rupture their membranes and 4 women with missing data on membrane rupture were excluded (n = 240). Additionally, 1/25 HPV positive infant was excluded from this analysis since the baby was born by elective caesarean section (intact membranes). ^A^ Adjusted for age, ethnicity and smoking status. ^B^ Adjusted for age, ethnicity, gestational age at delivery, parity, smoking status, household income, university education and new sexual partner. ^C^: No variable was found to have a confounding effect.

## Data Availability

The raw data supporting the conclusions of this article will be made available by the authors on request.
